# Biomarkers in biliary atresia – an elusive promise of predicting outcome

**DOI:** 10.1038/s41390-024-03772-6

**Published:** 2025-01-07

**Authors:** Barath Jagadisan, Anil Dhawan

**Affiliations:** Pediatric Liver GI and Nutrition Centre and Mowat Labs, King’s College Hospital NHS Foundation Trust, London, UK

## Abstract

Research on biomarkers in BA promises to predict and change outcomes in BA. This goal is still unrealized at the moment. The study by Kamp et al attempts to understand the drivers of fibrosis and elucidate the role of amyloid-related genes in the pathophysiology of BA. Based on this, they attempt to predict outcomes in BA using biomarkers by analysing plasma samples for amyloid precursor protein. There is a need to evaluate if shifting the focus from pre-KPE biomarkers to post-KPE biomarkers, serial evaluations and the use of composite scores could help in better prognostication.

Biliary atresia (BA) is the most common cause of chronic liver disease progressing to liver transplantation (LT) in children.^1^ Among patients with BA who do not clear jaundice within 3 months following the Kasai portoenterostomy (KPE) surgery, the disease usually progresses leading to liver decompensation.^2,3^ Those who survive beyond 2 years of age with a native liver, still continue to have disease progression resulting in only 28% of children with BA entering adulthood with their native liver.^4^ Timely KPE, proactive nutritional management and aggressive management of cholangitis are the factors that usually impact the outcome.^5–14^ Hence a better understanding of the pathophysiological process leading to the worsening of liver disease could help the management and influence the outcomes.

The study by Kamp et al. in this issue of Pediatric Research attempts to understand the drivers of fibrosis and elucidate the role of amyloid-related genes in the pathophysiology of BA. They use targeted gene profiling of a commercial panel of 760 fibrosis-specific genes and eight bile acid-related genes in liver biopsy specimens obtained at the time of KPE. Based on this, they attempt to prognosticate BA using biomarkers by analysing plasma samples for amyloid precursor protein (APP) as a biomarker in 30 BA infants and 10 age-matched controls.^15^ Biomarkers have been studied in BA for screening, diagnosis, prognostication of native liver survival, estimation of degree of fibrosis and clinically significant portal hypertension. Neonatal serum conjugated bilirubin measurement has been used to screen for BA.^16^ To avoid invasive investigations for diagnosis in infants with cholestasis, serum biomarkers such as Gamma-glutamyl transpeptidase and Matrix metalloproteinase-7 (MMP-7) have been extensively studied but with limited clinical application. The variable cut-offs between studies and differences in the performance of the individual kits limit the use of MMP-7.^17,18^ Ideally, clinically relevant biomarkers should approach a 100% negative predictive value for diagnosing BA to be translated to clinical practice. However, given the time constraints, acholic stools, abnormal gall bladder, liver biopsy and peroperative cholangiogram remain the mainstay of diagnosis. Biomarkers are likely to be more helpful in informing treatment choices and prognosis in BA.

Kamp et al. show gene clustering associated with different fibrosis stages, without clustering for the various clinical outcomes [infants who survived with native liver but had recurrent jaundice (*n* = 3), infants who survived jaundice-free with native liver (*n* = 3), infants underwent LT - early LT within one year after KPE (*n* = 4) and LT after the first year after KPE, (*n* = 4)]. Elevated expression of APP was noted. Genes associated with amyloidosis (ADAM9, APOA1, APOA2, APP, LPR1, MMP7, MMP14 and PSEN2) also showed clustering with different fibrosis stages without this clustering in clinical outcomes. APP staining was demonstrated in BA livers mainly in the biliary epithelial cells or bile duct lumen and also in periportal hepatocytes occasionally.^15^ These findings are consistent with the fact that serum Amyloid P component (SAPC) has been shown to have immune regulatory functions in the liver.^19^ SAPC levels are known to be decreased in other liver diseases such as models of metabolic dysfunction associated steatohepatitis.^20^ APP as a plasma biomarker showed no correlation with clinical outcomes but only with the various stages of fibrosis.^15^ It is not clear if all these samples are from the time of KPE or from time points after KPE. The time point of sampling for biomarkers is essential in being able to interpret the results.

The categorisation of outcomes into the 4 groups by Kamp et al tends to decrease the sample size into small groups, which makes analysis and interpretation challenging.^15^ The major challenge faced by the treating team is identifying the patient to whom KPE should not be offered, even without liver synthetic dysfunction or other evidence of decompensation. They have traditionally been identified either by using age at diagnosis as the predominant criterion or by the presence of advanced fibrosis. It is known that neither age nor the biopsy at the time of KPE reliably identify these patients. Liver histology at the time of KPE is reported for the grade of fibrosis, the degree of portal inflammation and the ductular remnant size, but these features also do not predict the outcome of the KPE.^5,14,21–29^ The confounding effects of heterogeneity of the disease, surgical expertise in individual studies and dynamic changes post-KPE including cholangitis cannot be overstated. The sampling variability of liver biopsies and the overestimation of fibrosis in surgical wedge biopsies add a layer of complexity to interpretation. Despite evaluating several biomarkers, reliable case selection for KPE with a single pre-surgical biomarker has not been possible.^17,18^

Infants who demonstrate sufficient biliary drainage as evidenced by the biomarker of total serum bilirubin <2 mg/dL at 3 months post-KPE have a better 2-year transplant-free survival.^2^ The factors contributing to the progression of disease are poorly understood. Still, they could include pre-existing architectural distortion of the extracellular matrix and biliary system that impair bile drainage irrespective of the KPE, ongoing biliary injury from inflammation, variations in the surgical drainage depending on the technique and recurrent episodes of cholangitis. Biomarkers in these patients could help (a) determine the efficacy of bile drainage, (b) diagnose and treat cholangitis, (c) identify clinically significant portal hypertension and (d) predict the prognosis for transplant-free survival. The biomarkers of prognosis define subpopulations of BA that are homogenous in their outcomes, thereby providing a means to risk-stratify groups for interventional studies that attempt to modify outcomes. With a better understanding of the pathophysiology and availability of interventions targeting specific pathophysiological processes, biomarkers could define subgroups homogenous in their pathophysiology.

Among several biomarkers studied recently to predict fibrosis or native liver survival in BA, MMP-7 has attracted much attention. However, except for one study, most studies have found a correlation between MMP-7 and liver fibrosis or stiffness.^30–33^ The correlation of changes in the levels of MMP-7 from before KPE to different time points after KPE to the clinical outcomes has again yielded contradictory results.^32,34,35^ Mac-2 binding protein glycan isomer (M2BPGi), secreted by hepatic stellate cells, has been shown to predict severe grades of fibrosis.^36,37^ Serial estimations of M2BPGi may predict worsening fibrosis and liver dysfunction.^38^ Other biomarkers studied to predict fibrosis or native liver survival include fibroblast growth factor-19, mac-2 binding protein glycan isomer, interleukin-13Rα2, aspartate aminotransferase to platelet ratio index, cartilage oligomeric matrix protein, long non-coding RNA-H19, microRNA-214, serum total bile acids, Hyaluronic acid, interleukin-8, interleukin-18 and interleukin-12p40.^17,18^ The study by Kamp et al adds APP to the list of candidate biomarkers for prognostication but will need validation in a larger sample of post-KPE patients.^15^ Wider definitions of biomarkers consider transient elastography measurements of the liver as a biomarker.^39^ Very early liver stiffness measurements (LSM) after KPE are associated with the occurrence of thrombocytopenia, splenomegaly, and oesophagal varices 6 months post-KPE. The need for LT was higher in BA subjects with LSM > 16 kPa measured 1-week post-KPE than other BA subjects (hazard ratio=10.16; P = 0.04).^40^

Serum bile acids have emerged as a useful biomarker of biliary drainage in those with normalised serum bilirubin. Serum bile acids >40 μmol/L at six months after KPE in these patients predicts the development of portal hypertension and synthetic dysfunction at 2 years of age. Those with serum bile acid levels ≤40 μmol/L had a lower 10‐year cumulative incidence of LT/death.^41^ Apart from being a prognostic marker or a risk-stratifying parameter for interventional studies, it remains to be seen if serum bile acid level would be a relevant surrogate therapeutic target to improve outcomes.

Recurrent cholangitis is seen as a major predictor of outcomes by potentiating fibrosis and liver dysfunction, except in a few studies.^42–48^ There is a need for biomarkers that can reliably identify cholangitis and also guide the duration of the antibiotic course.^49–52^ It is necessary to acknowledge that the outcome in BA is influenced by a combination of factors, including fibrotic architectural distortion, ongoing biliary injury, recurrent cholangitis and impaired biliary drainage. Assessment of prognosis or stratification of at-risk groups entirely based on a single parameter like APP, reflecting only fibrosis or inflammation is unlikely to reflect the whole pathobiology. It would be worthwhile to interrogate if composite scores that reflect the presently understood pathobiology can outperform individual biomarkers. Also, the trajectory of the patient’s clinical course can be altered by events such as cholangitis and the resulting disruption of biliary drainage. Hence, serial estimation of biomarkers interpreted in conjunction with clinical parameters will probably help in a better understanding of the progression of disease in BA.

The study by Kamp et al. is a step towards understanding the mechanism of fibrosis and developing biomarkers for prognostication in BA.^15^ Research on biomarkers in BA comes with the promise of changing outcomes in BA. This goal is still unrealised at the moment. There is a need to evaluate if shifting the focus from pre-KPE biomarkers to post-KPE biomarkers, serial evaluations and the use of composite scores could help in better prognostication.
